# Myxoid liposarcoma: it's a hippo's world

**DOI:** 10.15252/emmm.201910470

**Published:** 2019-04-12

**Authors:** Carla Regina, Simone Hettmer

**Affiliations:** ^1^ Division of Pediatric Hematology and Oncology Department of Pediatric and Adolescent Medicine University Medical Center Freiburg University of Freiburg Freiburg Germany

**Keywords:** Cancer

## Abstract

Fused in sarcoma: DNA damage‐inducible transcript 3 protein (FUS:DDIT3) is a chimeric fusion oncoprotein present in 90% of human myxoid liposarcomas (MLS). The study by Trautmann *et al* in this issue of *EMBO Molecular Medicine* utilizes a drop‐out RNAi screen to establish hyperactive Yes‐associated protein 1 (YAP1), a major downstream nuclear effector of the Hippo signaling pathway, as a selectively essential transcript promoting viability and growth of MLS. These observations add to a growing body of evidence underscoring the importance of dysregulation of Hippo signaling in soft‐tissue sarcomas expressing fusion oncoproteins and identify a novel target for therapeutic intervention in MLS. Comprehensive molecular characterization pipelines are needed to screen patients with advanced soft‐tissue sarcomas for the presence of druggable alterations, including but not limited to nuclear YAP1 expression in MLS, to facilitate treatment decisions and advance therapy.

Fusion genes are common types of oncogenes in human cancers, resulting in new, hyperactive combinations of regulatory elements and functional protein domains. The discovery of fusion oncogenes in the 1970s stimulated the development of potent therapeutics such as imatinib, a tyrosine kinase inhibitor for the treatment of myeloid leukemias expressing the breakpoint cluster region: abelson murine leukemia viral oncogene homolog 1 (BCR:ABL1) kinase fusion. Imatinib was coined as a magic bullet to treat cancer. Since then, dozens of other fusion oncoproteins have been identified in hematological cancers, tumors of the central nervous system, carcinomas, and sarcomas. Many of them are tumor‐specific and provide immense diagnostic value due to their inherent expression in certain tumor types. The fusion oncogene fused in sarcoma: DNA damage‐inducible transcript 3 protein (FUS:DDIT3) originates from a chromosomal translocation t(12;16) (q13;p11) in 90% of human myxoid liposarcomas (MLS), belongs to the FET (FUS, EWSR1 and TAF15) family of chimeric oncoproteins, and contains the N‐terminal domain of FUS juxtaposed to the DNA binding domain of the transcription factor DDIT3. FET‐family and many other transcription/chromatin factor fusions are typically associated with low tumor mutational burdens, occur early in tumorigenesis to initiate and drive malignancy, and should qualify as ideal targets to selectively attack fusion‐positive tumor cells. Yet, their discovery in leukemias and sarcomas has not translated into improvements in treatment and survival rates, because they act as transcriptional dysregulators and successful pharmacological modulation using small molecules has not been possible thus far (Parker & Zhang, 2013).

Trautmann *et al* (2019) argue that the identification of signaling pathways, that are essential in cells expressing chimeric fusion oncoproteins, represents a highly promising strategy to selectively target these cancer cells. Their present study (Trautmann *et al*, 2019) utilized a drop‐out RNAi screen in FUS:DDIT3‐expressing, immortalized human mesenchymal stem cell lines as an unbiased functional genomic approach to reveal that FUS:DDIT3‐expressing cells require Yes‐associated protein 1 (YAP1), a downstream nuclear effector of the Hippo signaling pathway, to maintain viability and cell growth. Among liposarcoma cell lines, YAP1 expression was detected in one pleomorphic and three MLS cell lines. Moreover, YAP1 was shown to primarily localize to the nucleus of MLS cells by immunohistochemistry and nuclear fractionation, indicating transcriptional activity as co‐activators of downstream targets such as forkhead box M1 (FOXM1) and polo‐like kinase 1 (PLK1). Moderate or strong nuclear YAP1 expression was confirmed in 77 (91%) of 85 primary MLS samples, and YAP1 co‐localized with FUS:DDIT3 in the nucleus of MLS cells, indicating that YAP1 and FUS:DDIT3 closely cooperate to orchestrate a gene expression program that promotes MLS development (Fig. 1). In fact, silencing of YAP1 resulted in G1 arrest, senescence, and apoptosis, supporting a dependence on YAP1 of FUS:DDIT3‐positive MLS cell lines. Trautmann *et al* (2019) went on to further examine the relationship between FUS:DDIT3 and increased YAP1 activity in MLS by overexpressing FUS:DDIT3 in human mesenchymal stem cell lines. This led to expression, nuclear translocation, and activation of YAP1 and of its effectors PLK1 and FOXM1. Importantly, Trautmann *et al* (2019) also demonstrated that YAP1 may serve as a novel target for therapeutic intervention, as chemical inhibition and shRNA‐mediated knockdown of YAP1 suppressed viability, proliferation, and growth on the surface of chick embryo chorioallantoic membranes (CAM) of MLS cell lines.

**Figure 1:**
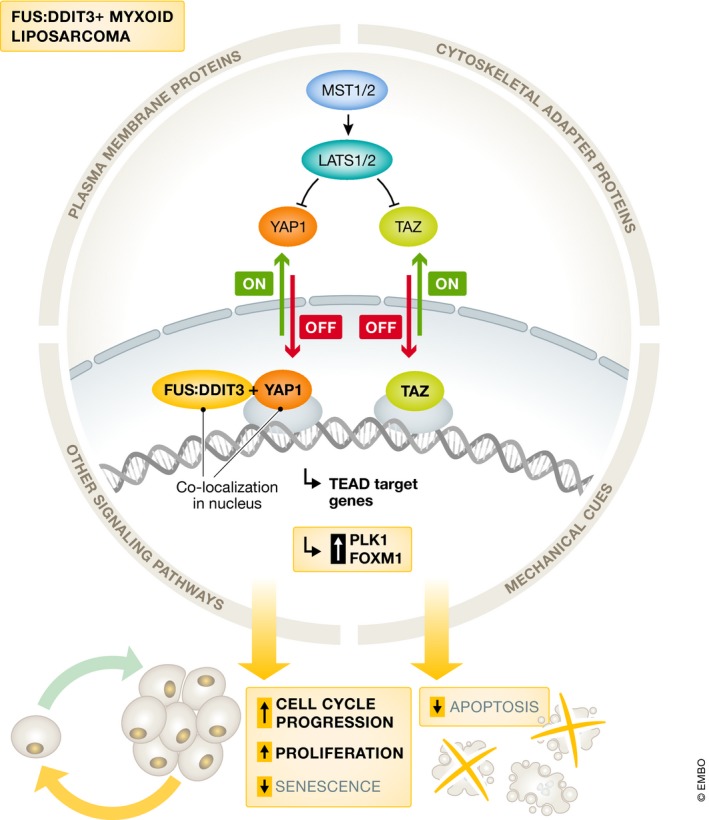
FUS:DDIT3 expressing cells require the Hippo effector YAP1 to maintain growth Canonical Hippo signaling involves serine/threonine kinases MST1/2 (which are homologs of Drosophila Hippo) and LATS1/2. The Hippo pathway is “on”, when MST1/2 and LATS1/2 kinases are active, which results in cytoplasmic retention and proteosomal degradation of YAP1 and TAZ. When Hippo signaling is “off”, YAP and TAZ localize to the nucleus and function as transcriptional co‐activators for TEA domain‐containing sequence‐specific and other transcription factors. Hippo signaling is governed by inputs from mechanical cues, plasma membrane proteins, cytoskeletal adapter proteins, and other signaling pathways. The chimeric fusion oncoprotein FUS:DDIT3, present in 90% of MLS, co‐localizes with YAP1 in the nucleus of FUS:DDIT3‐expressing cells, which mediates cell cycle progression, proliferation, evasion of senescence, and less apoptosis.

YAP1 is a transcriptional regulator, which operates under the control of the Hippo pathway, an evolutionary conserved developmental network integrating signals from plasma membrane proteins, upstream adapter proteins, other signal transduction pathways, and intrinsic/extrinsic mechanical forces to regulate cell fate/differentiation, tissue homeostasis, and organ growth/size. Active Hippo signaling results in phosphorylation, cytoplasmic retention, and proteosomal degradation of the effector proteins YAP and tafazzin (TAZ). When Hippo signaling is inactive, for example during malignant transformation, YAP and TAZ localize to the nucleus, where they serve as transcriptional co‐activators for TEA domain‐containing sequence‐specific (TEADs) and other transcription factors in order to promote tumorigenic events (Fig. 1). Oncogenic mutations within the core components of the Hippo pathway are very rare, but suppressed Hippo transduction has been detected in many cancers, including various fusion‐positive soft‐tissue sarcomas such as alveolar rhabdomyosarcoma, epithelioid hemangioendothelioma, Ewing sarcoma, and, now, myxoid liposarcoma (Deel *et al*, 2015). In alveolar rhabdomyosarcoma, for example, high expression of cytoplasmic and nuclear YAP1 has been documented, and the fusion oncogene paired box gene 3 (PAX3): forkhead box protein O1 (FOXO1) directly regulates increased expression of Ras‐association domain family 4 (RASS4). RASS4 associates with mammalian STE20‐like protein kinase 1 (MST1) as an inhibitory complex to further reduce Hippo activation and thereby promotes cell cycle progression and evasion of cell senescence (Crose *et al*, 2014). In the present study, Trautmann *et al* (2019) show that FUS:DDIT3 co‐localizes with YAP1 in the nucleus of MLS cells, which may contribute to stabilization of active YAP1 in the nucleus. The mechanism by which FUS:DDIT3 acts to achieve/maintain YAP1 activation remains to be experimentally elucidated.

Recognition of the importance of Hippo signaling in malignancy has stimulated the development of pharmacological compounds that directly or indirectly modulate Hippo pathway activity. Several kinases in the Hippo network might serve as therapeutic targets, but most of them are tumor suppressors and restoring lost tumor suppressor function may prove to be difficult. Trautmann *et al* (2019) use verteporfin, a photosensitizer, which is in clinical use to treat patients with macular degeneration. Verteporfin binds to YAP1 altering its conformation and preventing it from binding to TEAD transcription factors (Deel *et al*, 2015). They show that verteporfin inhibits MLS growth *in vitro* and in chicken CAMs (Trautmann *et al*, 2019). However, a recent study revealed that verteporfin inhibited colon cancer growth by inducing imbalances in proteostasis and subsequent protein degradation (Zhang *et al*, 2015). Cyclic YAP‐like peptide or other chemical inhibitors of Hippo signaling (Deel *et al*, 2015) could thus help clarifying the results of the pharmacological assays. Furthermore, one should keep in mind that Hippo signaling is ubiquitously relevant, and systemic treatment may cause substantial side effects, especially in children and adolescents, in whom intact Hippo signaling is required for normal growth and development.

Liposarcomas account for 15–20% of all soft‐tissue sarcomas, and 20–30% are of the myxoid or myxoid round cell subtype. The latter arise preferentially in adolescents and younger adults, and they are characterized by a high rate of hematogenous metastases to extrapulmonary sites and/or recurrent tumors in up to 40% of patients. Local and/or distant failures typically take place within the first 5 years after diagnosis, but later events are not unusual. Surgery preceded or followed by radiation therapy represents the mainstay of treatment, resulting in 10‐year overall survival rates of approximately 70%. Although there is evidence to suggest that MLS tumors are chemotherapy‐sensitive, and despite the encouraging results in phase I/II studies of trabectedin, a marine‐derived novel anti‐cancer agent, neoadjuvant or adjuvant chemotherapy has never been associated with a clear survival benefit (Chowdhry *et al*, 2018). Treatment options for patients with inoperable or metastatic MLS continue to be poor, and the implementation of molecularly targeted therapies remains far behind. It has been reported that peroxisome proliferator‐activated receptor gamma (PPARγ) agonists such as rosiglitazone induce terminal differentiation of MLS cells *in vitro*, but phase II studies did not substantiate anti‐tumor effects in patients with advanced disease (Debrock *et al*, 2003). Hope for future improved therapies builds on published studies that have provided important insights into druggable alterations in MSL signaling: Activating mutations in the catalytic subunit of phosphatidylinositol‐4,5‐bisphosphate 3‐kinase catalytic subunit alpha (PIK3CA) occur predominantly in the more aggressive round cell variant of MLS (Trautmann *et al*, 2017). Moreover, myxoid liposarcomas were associated with FUS:DDIT3‐driven induction of insulin‐like growth factor 2 (IGF2) expression and activation of insulin‐like growth factor 1 receptor (IGF‐IR)/phosphoinositide 3‐kinase (PI3K)/AKT serine/threonine kinase 1 (AKT) signaling (Trautmann *et al*, 2017), overexpression of fibroblast growth factor receptor 2 (FGFR2) (Künstlinger *et al*, 2015), and activation of casein 2 kinase/atypical nuclear‐factor kappaB signaling (Willems *et al*, 2010). These observations corresponded to significant *in vitro* and/or *in vivo* inhibition of MLS growth after treatment with PI3K/mTOR inhibitors, IGF‐IR receptor inhibitors, FGFR inhibitors in combination with trabectedin, and the casein kinase 2 inhibitor 4,5,6,7‐tetrabromobenzotriazole (TBB) combined with dasatinib (Willems *et al*, 2010; Künstlinger *et al*, 2015; Trautmann *et al*, 2017). The present study by Trautmann *et al* introduces overactive YAP1 signaling as a hallmark of human MLS, and another candidate target for therapeutic intervention. To rapidly advance therapy and facilitate treatment decisions, it will be critical to implement comprehensive molecular characterization pipelines to screen patients with advanced soft‐tissue sarcomas, including MLS, for the presence of druggable alterations (e.g., nuclear YAP1, PIK3CA mutations, FGFR2 overexpression) and thereby identify candidates for early phase clinical trials.
